# The value of translational biomarkers to phenotypic assays

**DOI:** 10.3389/fphar.2014.00171

**Published:** 2014-07-15

**Authors:** David C. Swinney

**Affiliations:** Institute for Rare and Neglected Diseases Drug Discovery, Mountain ViewCA, USA

**Keywords:** drug discovery, phenotypic screening, molecular mechanism of action, MMOA, biomarkers, target-based

## Abstract

Phenotypic assays are tools essential for drug discovery. Phenotypic assays have different types of endpoints depending on the goals; (1) empirical endpoints for basic research to understand the underlying biology that will lead to identification of translation biomarkers, (2) empirical endpoints to identify undesired effects related to toxicity of drug candidates, and (3) knowledge-based endpoints (biomarkers) for drug discovery which ideally are translational biomarkers that will be used to identify new drug candidates and their corresponding molecular mechanisms of action. The value of phenotypic assays is increased through effective alignment of phenotypic assay endpoints with the objectives of the relevant stage in the drug discovery and development cycle.

## INTRODUCTION

The goal of the paper is to provide awareness that a key feature of phenotypic assays for drug discovery is the relationship of the measured endpoint to a biomarker that translates to the desired clinical response. In the early research phase phenotypic assays can be used to increase understanding of the disease and to identify potential translational biomarkers, while in the application phase in which the underlying knowledge of the disease is translated to treatments phenotypic assays should be aligned with translational biomarkers. Examples of drug discovery strategies show that the phenotypic endpoints for many of the successful strategies used previous knowledge that effectively translated to clinical outcomes.

Phenotypic assays measure a phenotype in a physiological system. The term “phenotypic assay” includes all preclinical assay formats that use physiological systems, e.g., animals, cells, and biochemical pathways. Phenotypic assays make few assumptions as to the molecular details of how the system works and provide an empirical method to probe effects in physiological systems. The phenotype most relevant to the practice of drug discovery is a phenotype that directly translated to the clinical disease (translational biomarker).

Phenotypic assays have always played an important role in drug discovery. Much of early pharmacology and drug discovery was based on phenotypic assays. Phenotypic assays were used to identify leads that provided the desired efficacy. In his nobel lecture entitled “Selective inhibitors of dihydrofolate reductase” George H. Hitchings Jr. stated “Those early, untargeted studies led to the development of useful drugs for a wide variety of diseases and has justified our belief that this approach to drug discovery is more fruitful than narrow targeting”(Hitchings, 1988). In the last decades of the 20th century the emphasis of drug discovery changed to a more reductionist, target-based approach and phenotypic assays were primarily used to confirm efficacy and evaluate safety. The hope was that the molecular and genetic revolution would provide numerous new medicines due in part to the capabilities to identify many new drug targets. Though not explicitly stated, the idea was that the drug targets would be biomarkers for the disease. Accordingly, a new paradigm of drug discovery emerged in which the target was the biomarker for disease. In this paradigm the central features are (1) identification of a molecule that binds to that target and (2) optimization of the biopharmaceutics properties such that the drug concentrations in the body are sufficient to ensure that the drug is bound to the target through-out the dosing interval. This target-based paradigm was envisioned to provide a more rational approach to drug discovery, analogous to a design and engineering approach. It is well documented that this approach has not produced the desired results and in fact productivity has dramatically decreased (Scannell et al., 2013).

Phenotypic assays in animals have always been required to evaluate the safety of a drug substance. In the last few years there has been a reemergence of interest in using phenotypic assays to drive discovery. Swinney and Anthony (2011) analyzed the discovery strategies for new molecular entities (NMEs) that were approved by the U.S. Food and Drug Administration (FDA) between 1999 and 2008. Of the 259 agents that were approved, 75 were first-in-class drugs with new molecular mechanism of action (MMOAs), and out of these, 50 (67%) were small molecules and 25 (33%) were biologics. The results also showed that the contribution of phenotypic screening to the discovery of first-in-class small-molecule drugs exceeded that of target-based approaches—with 28 and 17 of these drugs coming from the two approaches, respectively—in an era in which the major focus was on target-based approaches. A more recently analysis by Swinney and Xia (2014) of the 102 NMEs approved between 1999 and 2012 for rare diseases showed a similar trend of success with phenotypic strategies; for first in class NMEs there were 15 that used phenotypic drug discovery (PDD), 12 that used target-based drug discovery (TTD) and 18 for biologics.

What is required to realize the full value of PDD in the 21st century? There are many aspects that are important including the quality of the assays, the sources of drug substance and the strategies to move compounds forward through development despite incomplete knowledge of their mechanisms of action. Another important feature is the choice and predictability of the phenotypic endpoint, which is the focus of this short report.

## THE DRUG DISCOVERY AND DEVELOPMENT CYCLE

**FIGURE 1:**
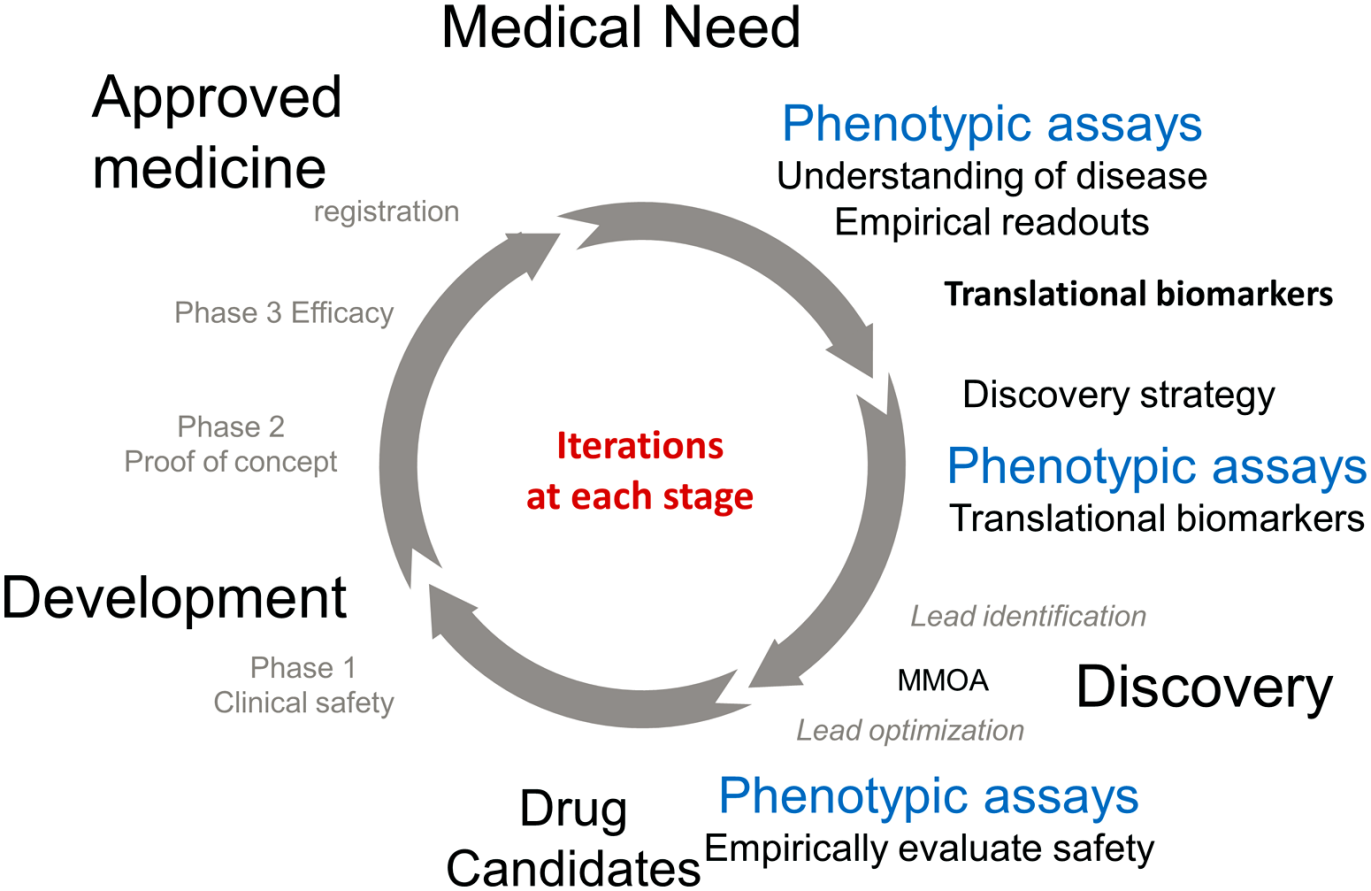
**Drug discovery research and development cycle**.

The approval of a medicine to treat an unmet medical need involves an iterative cycle of testing and learning. **Figure 1** describes some of the important phases in the process. The process of discovery and development of a new medicine is initiated in response to an unmet medical need to treat a disease. Physiological, genetic, and chemical knowledge provide an understanding of the disease. This knowledge will lead to the identification of translation biomarkers that are used to evaluate the effectiveness of a potential medicine. This is the research phase (12–3 o’clock). Phenotypic assays run in the research phase are extremely important to the understanding of the underlying biology and to help identify translational biomarkers.

The knowledge obtained in the research phase is used to inform the discovery phase (3–6 o’clock). The available knowledge informs drug discovery strategies which are used as starting points for the practical process of discovering a new medicine. TDD is associated with modulating a specific gene product known as the target, PDD is a strategy driven by assays which measure phenotypes associated with the disease. Ideally these phenotypes will be the associated with the translational biomarkers. These two strategies are primarily focused on small molecules and are medicinal chemistry intensive, in contrast to biologics which use recombinant proteins and antibodies as therapeutics. It should be noted that the knowledge to choose a strategy is generally incomplete, however the more iterations that occur in the drug discovery/development cycle the more complete the knowledge and the better chance that a molecule will make it to registration. The discovery strategies will result in a lead molecule, ideally with activity against the translational biomarker. The molecule will work by a MMOA that provides an optimal therapeutic index. These molecules will then be optimized for biopharmaceutics properties and safety to provide a drug candidate. At this point the process of drug discovery is complete and the molecule should succeed or fail based on its own merit.

The left hand of the circle (from 6 to 12 o’clock) is the development phase of drug discovery which involves testing for safety and efficacy in humans leading to registration. Multiple iterations are generally required before a medicine with sufficient efficacy at a safe dose is discovered, tested in humans, and registered.

## HYPOTHESIS, MMOA, AND BIOMARKERS

Phenotypic assays have always been required to evaluate safety and recently, their value to identify efficacious molecules and their mechanisms of action for first-in-class medicines has been reevaluated (Swinney and Anthony, 2011; Kotz, 2012; Lee et al., 2012; Lee and Berg, 2013; Swinney, 2013a; Swinney and Xia, 2014). A major challenge in the identification of safe medicines is to identify MMOAs that provide both sufficient efficacy and safety (Swinney and Anthony, 2011; Swinney, 2011, 2013a). These MMOAs could be thought of as “pharmacological hot spots.” Due to the dynamic complexity of physiology both at the molecular and systems level it is difficult to *a priori* predict the exact interactions and molecules that will elicit a safe, therapeutically useful response, Empirical phenotypic assays provide an unbiased approach to identify the “pharmacological hot spots.”

In our earlier work we noted that in the target-based approach, drug discovery is generally hypothesis-driven, and in this case, there are at least three hypotheses that must be correct to result in a new drug (Swinney and Anthony, 2011). The first hypothesis applies to all discovery approaches: the hypothesis that activity in the preclinical screens used to select a drug candidate will translate effectively into clinically meaningful activity in patients. The other two related hypotheses are that the target selected is important in human disease and that the MMOA of drug candidates at the target in question is one that is capable of achieving the desired biological response. Successful first-in-class, TDD requires the time and resources to investigate all three hypotheses, and in particular, the importance of hypothesis-testing to identify an appropriate MMOA may be an underappreciated challenge, that – if neglected –could contribute to increased attrition for such approaches. In other words, it is clearly difficult to rationally identify the specific molecular interactions, from all the potential dynamic molecular interactions, that will contribute to an optimal MMOA. Thus the key biochemical nuances important for translation of the molecular interaction between a drug and the target to an optimal pharmacological response could be missed with target-based approaches. One value of the phenotypic approach is the unbiased identification of the MMOA.

## PHENOTYPIC ASSAYS FOR DRUG DISCOVERY

The endpoints for phenotypic assays can be anything that can be accurately measured and range from a systems end point such as blood pressure and seizures, to specific biomarkers including blood cholesterol and glucose for hyperlipidemia and diabetes, respectively. Current technologies in genomics and high content analysis allow measurement of many different markers of activity. For drug discovery it is important to understand and differentiate if these markers are translational biomarkers related to the clinical disease.

A closer look at the discovery biomarkers for the 28 NME’s categorized as phenotypic in the analysis of how medicines were discovered by Swinney and Anthony (2011) is shown in **Table 1**. As previously reported 10 of the medicines were identified in animal studies (Swinney, 2013b). The phenotypic endpoints for the studies were well correlated with clinical indications. Levetiracetam, rufinamide, and zonisamide were identified in well-establish models for anti-convulsant activity and aripiprazole in dopamine dependent activity known to be associated with anti-psychotic behavior. Ziconotide was discovered in a model for pain and ranolazine in animal model measuring anti-anginal and anti-ischaemic effects. The endpoints for ezetimibe, nateglinide, and pemirolast were blood cholesterol, blood glucose, and cutaneous anaphylaxis, respectively. Nitisinone is used to treat tyrosineamia type 1 and was originally developed as a herbicide and repurposed for the rare disease when safety studies demonstrated an effect on tyrosine metabolism (Swinney and Anthony, 2011; Swinney, 2013b).

**Table 1:** Discovery biomarkers for phenotypic assays of NMEs approved by US FDA between 1999 and 2008 (Swinney and Anthony, 2011).

Generic name	Discovery biomarker/assay	MMOA
Aripiprazole	Dopamine sensitive assays in animals	Partial agonist D2 receptor
Azacitidine	Cell based assays show effects on differentiation	Irreversible
Caspofungin Acetate	Inhibition of glucan synthesis *in vitro*	Non-competitive
Cilostazol	Blood platelet aggregation	Inhibitor
Cinacalcet Hydrochloride	Increased in Ca+2 in bovine parathyroid cells	Allosteric activator
Daptomycin	Cytotoxicity in antimicrobia	Unknown
Docosanol	Viral infection assays	Unknown
Ezetimibe	Cholesterol lowering in animals	Transporter slow kinetics
Fulvestrant	Binding followed by animal studies	Antagonist induced degradation
Levetiracetam	Audiogenic seizure susceptible mice	Unknown
Linezolid	Random screening against bacterial disease in plants	Conformational trap
Lubiprostone		Unknown
Memantine	Originally identified in early 1960s as anti-diabetic	Uncompetitive fast kinetics
Hydrochloride		
Miglustat	Glycolipid biosynthesis in HL-60 cells	Reversible inhibitor
Nateglinide	Hypoglycemic effects in fasted normal mice	Fast kinetics
Nelarabine	Cell based assays required for activation	Chain terminator
Nitazoxanide	Antimicrobial	Redox/irreversible
Nitisinone	Compounds originally discovered in screening against plants	Irreversible
Pemirolast Potassium	IgE induced anaphylaxis in animals	Unknown
Ranolazine	Animal models	Unknown
Retapamulin	Antimicrobial assays against resistant organism	Allosteric inhibitor
Rufinamide	Animal anticonvulsant	Unknown
Sinecatechins/green tea	No screening herbal/evaluated in humans	Unknown
extract		
Sirolimus	Screened in antimicrobial assays	Conformation inhibition
Varenicline	Focused approach culminating in animal assays	Partial agonist Nicotinic receptor
Vorinostat	Cell based assay/cytodifferentiation	Enzyme inhibitor
Ziconotide	Intra-cerebral injection into mice	Ion channel equilibrium kinetics
Zonisamide	Animals models of epilepsy	Unknown

The phenotypic end points for those discovered using cell based assays provide examples where cell death was used as the phenotypic marker. These included azacitidine, daptomycin, linezolid, nelarabine, retapamulin, and sirulimus, all were approved for use as either anti-infective or anti-cancer therapies. Vorinostat was discovered by its ability to induce cytodifferentiation and growth arrest. The phenotypic markers for docosanol and cilostazol were viral replication and platelet aggregation, respectively. For the discovery of varenicline mesolimbic dopamine levels were measured and for fulvestrant the estrogenic effects.

The phenotypic marker for cinacalcet was an increase in calcium [Ca^+2^] in bovine parathyroid cells. The investigators were looking to agonize a calcium receptor. Miglustat was prepared and tested to interfere with glycoprotein synthesis and was repurposed for the Gaucher’s disease, a glycosphingolipid storage disorder.

The phenotypic readouts for all these NMEs were well validated markers of physiological functions. Cell death, anti-convulsant activity, calcium activation, platelet aggregation, viral replication, blood cholesterol, and glucose levels all translate to clinical disease. In the course of the iterative R&D cycle these biomarkers have become validated translational markers used to align drug discovery with clinical development (**Figure 1**).

## FUTURE TRENDS

The goal of drug discovery is to identify medicines that can benefit patients at safe doses. The challenge to achieve this goal is to identify medicines that will be safe and efficacious prior to testing in human studies, in preclinical studies. The major point highlighted in this short paper is the importance of translational biomarkers for PDD and to point out the difference between phenotypic assays that are used to investigate the underlying disease biology. Both are important, the research assays can be used to identify translational biomarkers and the discovery assays apply this knowledge to identify new medical treatments. There is a great need for validated translational biomarkers to guide drug discovery in order to identify safe and effective medicines prior to clinical evaluation. This is key to decreasing attrition and increasing productivity of pharmaceutical research.

The reality is that the more relevant the system is to physiology the better it will predict the clinical success. Associated with this complexity is the feasibility of obtaining predictive information. Phenotypic assays that translate effectively to human disease will always be required for the reasons described above, including the ability to identify an optimal MMOA and derisk safety. Unfortunately predictive phenotypic assays and relevant biomarkers are not available for most human diseases. One of the hopes for the genetic revolution was to identify specific genotypes, genes, and targets that could be used to guide preclinical drug discovery to identify new medicines. This approach has not been as widely successful as hoped. Aligning these efforts to identify translational biomarkers for phenotypic assays should increase the successful discovery of new medicines.

## Conflict of Interest Statement

The author declares that the research was conducted in the absence of any commercial or financial relationships that could be construed as a potential conflict of interest.

## ABBREVIATIONS

MMOA: molecular mechanism of action
PDD: phenotypic drug discovery
TDD: target-based drug discovery.

## References

[B1] Hitchings G. H. Jr. (1988). *Selective Inhibitors of Dihydrofolate Reductase.* Available at: http://www.nobelprize.org/nobel_prizes/medicine/laureates/1988/hitchings-lecture.pdf 10.1007/BF02624591 2654121

[B2] Kotz J. (2012). Phenotypic screening, take two. *Sci. Bus. Exch.* 5 1–3

[B3] Lee J. A. Berg E. L. (2013). Neoclassic drug discovery: the case for lead generation using phenotypic and functional approaches. *J. Biomol. Screen.* 18 1143–1155 10.1177/1087057113506118 24080259

[B4] Lee J. A. Uhlik M. T. Moxham C. M. Tomandl D. Sall D. J. (2012). Modern phenotypic drug discovery is a viable, neoclassic pharma strategy. *J. Med. Chem.* 55 4527–4538 10.1021/jm201649s 22409666

[B5] Scannell J. W. Blanckey A. Boldon H. Warrington B. (2013). Diagnosing the decline in pharmaceutical R&D efficiency. *Nat. Rev. Drug Discov.* 11 191–200 10.1038/nrd3681 22378269

[B6] Swinney D. C. (2011). Molecular mechanism of action (MMOA) in drug discovery. *Ann. Rep. Med. Chem.* 46 301–317 10.1016/B978-0-12-386009-5.00009-6

[B7] Swinney D. C. (2013a). Phenotypic, vs. target-based drug discovery for first-in-class Medicines. *Clin. Pharmacol. Ther.* 93 299–301 10.1038/clpt.2012.236 23511784

[B8] Swinney D. C. (2013b). The contribution of mechanistic understanding to phenotypic screening for first-in-class medicines. *J. Biomol. Screen.* 18 1186–1192 10.1177/1087057113501199 23983234

[B9] Swinney D. C. Anthony J. (2011). How were new medicines discovered? *Nat. Rev. Drug Discov.* 10 507–519 10.1038/nrd3480 21701501

[B10] Swinney D. C. Xia S. (2014). How medicines for rare diseases were discovered. *Future Med. Chem.* 6 (in press) 10.4155/fmc.14.65 PMC4354801 25068983

